# Exploring Patient Safety Culture: A Study of Community Pharmacies in Karachi, Pakistan

**DOI:** 10.7759/cureus.52135

**Published:** 2024-01-11

**Authors:** Shoaib A Khatri, Rabbiya Ahmad, Muhammad Osama, Kamran Khan, Masood Ahmed Khan, Azfar Ishaqui, Narendar Kumar

**Affiliations:** 1 Pharmacy, Sindh Government Hospital, Karachi, PAK; 2 Clinical Pharmacy, School of Pharmaceutical Sciences, Universiti Sains Malaysia, Gelugor, MYS; 3 Pharmacy, University of Karachi, Karachi, PAK; 4 Pharmacy, Institute of Pharmaceutical Sciences, Jinnah Sindh Medical University, Karachi, PAK; 5 Pharmacy, DVAGO Pharmacy, Karachi, PAK; 6 Pharmacy, Iqra University, Karachi, PAK; 7 Pharmacy Practice, Faculty of Pharmacy, University of Sindh, Jamshoro, PAK

**Keywords:** pharmacist care, observational cross-sectional study, pakistan, community pharmacy, patient safety

## Abstract

Background

Community pharmacies are integral to the healthcare system, actively contributing to patient safety through accurate dispensing, education, collaboration, monitoring, and the implementation of safety protocols. Their accessibility and role as medication experts make them key partners in promoting positive health outcomes for individuals and communities.

Objective

The current study will evaluate the patient safety culture (PSC) among community pharmacies in Karachi, Pakistan. Additionally, this study will measure the association between patient safety culture in community pharmacies and the demographic characteristics of the pharmacy staff.

Methods

A cross-sectional survey of pharmacy staff was conducted using a survey instrument developed by the US Agency for Healthcare Research and Quality (AHRQ). Demographic variables and assessments of safety culture in pharmacies were studied. The data were analyzed using descriptive statistics.

Results

Among the 102 participants, positive responses ranged from 30% to 87.5%. The highest positive response was for the dimension "mistakes in communication" (86.3%), followed by "communication across shifts" (82.2%) and "communication openness" (81.7%). The dimensions "overall perceptions of patient safety" and "response to mistakes" had the lowest positive responses (56.0% and 60.9%, respectively). Furthermore, many staff did not regularly record the errors, even if they impacted the practices.

Conclusion

There was an overall unfavorable perception of patient safety culture among the surveyed pharmacies of Karachi, Pakistan. However, the communication dimensions showed the highest positive response. There is a strong need to improve the overall perception of patient safety among the staff and develop an optimistic response to mistakes.

## Introduction

Patient safety forms the cornerstone of high-quality healthcare. Patient safety, as defined by the World Health Organization (WHO), involves the proactive prevention of mistakes and adverse outcomes in healthcare, with a primary focus on avoiding harm to patients [1]. One of the key pillars for maximizing patient safety is encouraging a "culture of safety" inside healthcare organizations. Unfortunately, a significant number of patients are injured during the provision of healthcare services, resulting in permanent disability, prolonged hospitalization, or even death [2]. Therefore, the mounting interest has encouraged patient safety research to explore organizational features that affect patient safety. One such feature is patient safety culture (PSC), a shared set of beliefs and values that shape the behavior of individuals and organizations and persistently strive to minimize patient harm resulting from the care delivery process [3].

Medication safety is an important global concern, and it is monitored and assessed using pharmacovigilance systems. Medication errors are common, with 1.5 million medication errors occurring each year in the United States, a rate of 171 errors per hour [3,4]. While much of the focus on medication errors has been on hospital settings, medication errors in nonhospital settings are also a concern. A recent study by Hong et al. reviewed medication errors reported to poison control centers across the United States that were outside of hospital settings and resulted in serious medical outcomes [4]. Community pharmacies offer a vital function within medication management in primary care, amalgamating numerous errands linked to medicine (including but not limited to dispensing medications and counselling the patients) with the necessity to function as sustainable commercial businesses [5]. The provision of community pharmacy services has turned out to be progressively challenging and complex; however, the way safety is produced and enacted in community pharmacy settings in this complexity remains inadequately understood [4,6]. However, a deep understanding of PSC in community pharmacies can promote an organization's quality improvement efforts by implementing strategies to enhance employee awareness of patient safety practices and conducting assessments to identify areas of strength that may require improvement.

Despite being regulated by the Drug Regulatory Authority of Pakistan (DRAP) Act 2012, the state of pharmacy services is inadequate, with limited availability of primary services, including the distribution, management, and dispensing of medications at retail pharmacies, which results in a lack of research studies examining the community pharmacy services in the country [7]. Historically, the provision of patient-centered pharmacy services and the availability of registered pharmacists have been marred by deficiencies at various levels in the country [8]. This study seeks to address this gap by investigating the patient safety culture within community pharmacies in Karachi, Pakistan. The research aims to explore the association between PSC and the demographic characteristics of pharmacy staff, providing valuable insights that can contribute to enhancing patient safety practices in these settings. This investigation is crucial for understanding the complexities of safety implementation in community pharmacies and will serve as a foundation for targeted quality improvement efforts. Through this study, we aim to bridge existing knowledge gaps and pave the way for more effective patient-centered pharmacy services in Pakistan.

## Materials and methods

Study design and setting

This cross-sectional study, conducted between February and April 2023, aimed to assess the PSC among staff working in community pharmacies in Karachi, Pakistan, using a web-based, self-administered questionnaire. Karachi is a metropolitan city in Pakistan and the 12th biggest city in the world, with an estimated population of 16.1 million [9,10]. Community pharmacies in Karachi serve as accessible and convenient healthcare resources for individuals residing in the area, and they offer a range of essential retail pharmacy services, encompassing prescription handling, dispensing medications, patient counselling, and maintaining comprehensive records. These pharmacies play a vital role in delivering healthcare services and are instrumental in promoting patient safety.

Study participants

The study participants included pharmacists, trainee pharmacists, and technical staff employed in community pharmacies. The qualification for technical staff was a diploma in pharmacy, which is a two-year program after high school. Only individuals aged 18 years and older who had a proficient understanding of English were included in the study. The participants were recruited using a convenience sampling technique, whereby individuals who met the eligibility criteria and were readily available were approached to participate.

Study instrument

The assessment of safety culture encompassed various aspects of patient safety, utilizing the patient safety culture survey developed by the US Agency for Healthcare Research and Quality (AHRQ) [11]. These aspects included patient counselling, communication openness, patient acuity, teamwork, organizational learning, improvement, effective communication regarding prescriptions across shifts, communication regarding mistakes, response to mistakes, staff training and skills, physical environment, and appropriate staffing and workload. This survey instrument consisted of 36 elements that assessed 11 aspects of organizational culture related to patient safety. The demographic section of the survey instrument was modified to suit the study population in Karachi, Pakistan. The survey was developed using Google Forms (Google, Inc., Mountain View, CA), and a unique link was generated to ensure participant access. The estimated time for survey completion ranged from 10 to 15 minutes. All the answers were mandatory to fill in as the questionnaire was designed this way. There was no option to skip any question.

The survey instrument included questions related to the participants' demographic characteristics (gender, age, experience, working hours per week, and position in the pharmacy), followed by questions addressing various aspects of PSC. The survey responses were measured in two ways: 1) a three-point agreement scale ranging from "disagree" (one point) to "agree" (three points) or 2) a three-point frequency scale ranging from "never" (one point) to "always" (three points). The survey questionnaire included a question where the participants were asked to rate the level of patient safety in their respective pharmacies using a scale that ranged from poor to excellent. The participants were encouraged to provide their subjective assessment of the patient safety standards observed in their pharmacy environments. To evaluate the positive response rate (PRR) for positively worded elements, the calculation involved determining the percentage of participants who responded with "agree," "always," or "most of the time." This percentage was then divided by the total number of participants to obtain the PRR. Likewise, for negatively worded items, the calculation involved determining the percentage of participants who responded with "disagree," "never," or "rarely." This percentage was then divided by the total number of participants to obtain the corresponding percentage for the negative response rate.

Data collection

In this web-based study, a self-administered survey was conducted using an Android tablet provided by the authors. Upon clicking the survey link, the participants were presented with the option to give their informed consent and proceed to other subsequent sections of the survey independently. Before starting the questionnaire, the study participants were comprehensively briefed regarding the primary purpose and content of the survey. Notably, participation in this study was voluntary, and the participants were not obligated to complete the questionnaire. The survey instrument underwent rigorous pilot testing to ensure standardization and comparability of the outcomes across different pharmacies and regions. However, the responses collected during the pilot study were not incorporated into the final analysis to maintain integrity.

Ethical approval for this research was sought from the Institutional Bioethics Committee (IBC) of the University of Sindh, Jamshoro, Pakistan (reference number: ORIC/SU/1336). Stringent measures were implemented throughout the data collection process to uphold anonymity and confidentiality. Special care was taken to ensure that participant identities and responses remained anonymous and strictly confidential. Before conducting the study in the pharmacies, verbal consent was obtained from the pharmacy services manager and the participants, indicating their understanding of the research objectives and willingness to participate.

Data analysis

The data analysis was conducted using the Statistical Package for Social Sciences (SPSS) version 27 software (IBM SPSS Statistics, Armonk, NY). Categorical variables were represented in terms of frequencies and percentages, while continuous variables were described using mean values and standard deviations (SD). The results were expressed as percent positive response (PPR) for individual elements and composites. The association between categorical variables was assessed using the chi-squared test. A p-value of <0.05 was considered statistically significant.

## Results

Sociodemographic characteristics of the participants

Out of 106 community pharmacy staff members approached for the study, 102 respondents participated, resulting in a response rate of 96.2%.

Table 1 displays the sociodemographic characteristics of the participants. Most participants were female (n = 69, 67.6%), with a mean age of 26.58 ± 3.32 years, with less than one year of professional experience in retail pharmacies (n = 44, 43.1%). Moreover, the pharmacy working hours were more than 40 per week in around three-fourths of the study participants (72.5%).

**Table 1:** Participants' sociodemographic characteristics (N = 102) *Pharmacy technician and managerial staff SD: standard deviation

Variables	n (%) or mean ± SD
Gender	
Female	69 (67.6)
Male	33 (32.4)
Age in years	26.58 ± 3.32
Age groups	
18-25	50 (49.0)
>25-30	38 (37.3)
>30	14 (13.7)
Professional experience in the facility	
<1 year	44 (43.1)
1-3 years	42 (41.2)
>3 years	16 (15.7)
Working hours per week	
<30	22 (21.6)
30-40	6 (5.9)
>40	74 (72.5)
Pharmacy position	
Pharmacy intern	16 (15.7)
Pharmacist	82 (80.4)
Others*	4 (3.9)

Assessment of safety culture across community pharmacies in Karachi

Table 2 represents the study participants' perceptions of PSC standards and pharmacy setup dimensions. The highest PRR score was for the dimension "communication of mistakes" (83.6%), followed by "communication openness" (80.4%), "communication across shifts" (80.4%), "patient counselling" (79.7%), and "teamwork" (77.1%). In general, the survey findings revealed a prevalence of positive responses rather than negative ones for the individual survey elements, and the positive responses ranged from 29.4% to 86.3%. The overall PRR for all 36 elements of the survey was calculated to be 72.11%. The detailed scores are presented in Table 2.

**Table 2:** Perception of the participants regarding PSC standards and dimensions (N = 102) PRR, positive response rate; PSC, patient safety culture

Individual items and dimensions	Negative response, N (%)	Neutral response, N (%)	Positive response, N (%)
Section A. Working in this pharmacy			
Environment and physical spacing (PRR = 68.6)			
This pharmacy setup is well-organized	4 (3.9)	18 (17.6)	80 (78.4)
This pharmacy setup is free of clutter	14 (13.7)	28 (27.5)	60 (58.8)
The overall layout of this pharmacy promotes good workflow	12 (11.8)	20 (19.6)	70 (68.6)
Teamwork (PRR = 77.1)			
Staff give respect to each other	8 (7.8)	16 (15.7)	78 (76.5)
All staff of this pharmacy understand their roles and responsibilities	2 (2.0)	26 (25.5)	74 (72.5)
Staff cooperate with each other and work as an effective team	4 (3.9)	14 (13.7)	84 (82.4)
Staff training and skills (PRR = 75.5)			
Pharmacy technicians receive the appropriate training they need to do their jobs	4 (3.9)	20 (19.6)	78 (76.5)
The staff of this pharmacy possess the skills they need to do their jobs well	8 (7.8)	18 (17.6)	76 (74.5)
Newly appointed staff receive an adequate orientation	4 (3.9)	20 (19.6)	78 (76.5)
Staff get enough training from this pharmacy	8 (7.8)	18 (17.6)	76 (74.5)
Section B. Communication and work pace			
Communication openness (PRR = 80.4)			
Staff suggestions and ideas are valued in this pharmacy	8 (7.8)	18 (17.6)	76 (74.5)
Staff are comfortable in asking questions about the things they are unsure about	14 (13.7)	4 (3.9)	84 (82.4)
Staff can easily talk to the supervisor/manager about patient safety concerns at this pharmacy	8 (7.8)	8 (7.8)	86 (84.3)
Patient counselling (PRR = 79.7)			
Patients are encouraged to talk to the pharmacists about their medications	10 (9.8)	18 (17.8)	74 (72.5)
Our pharmacists give adequate time to the patients about the use of their medications	16 (15.7)	4 (3.9)	82 (80.4)
Our pharmacists inform the patients about the necessary information of their new prescriptions	8 (7.8)	6 (5.9)	88 (86.3)
Staffing and work pressure (PRR = 68.4)			
Staff are given sufficient breaks during their shifts	16 (15.7)	16 (15.7)	70 (68.6)
Staff feel rushed when processing prescriptions	10 (9.8)	26 (25.5)	66 (64.7)
There is appropriate staffing to handle the workload	12 (11.8)	10 (9.8)	80 (78.4)
Distractions/interruptions (from phone calls, faxes, customers, etc.) in this pharmacy make it difficult for us to perform accurately	26 (25.5)	14 (13.7)	62 (60.8)
Communication across shifts (PRR = 80.4)			
We have clear expectations about sharing necessary information about prescriptions across shifts	8 (7.8)	8 (7.8)	86 (84.3)
There are standard procedures for communicating prescription information across shifts	8 (7.8)	18 (17.8)	76 (74.5)
The status of problematic prescriptions is communicated well across shifts	14 (13.7)	4 (13.7)	84 (82.4)
Communication of mistakes (PRR = 83.6)			
Staff discuss the mistakes	8 (7.8)	6 (5.9)	88 (86.3)
When patient safety problems occur in the pharmacy, staff discuss them	12 (11.8)	10 (9.8)	80 (78.4)
We discuss ways to prevent mistakes from reoccurring	8 (7.8)	6 (5.9)	88 (86.3)
Section C. Patient safety and response to mistakes			
Response to mistakes (PRR = 58.1)			
Staff are fairly treated upon committing mistakes	6 (5.9)	34 (33.3)	62 (60.8)
The pharmacy setup helps the staff to learn from their mistakes rather than punishing them	4 (3.9)	22 (21.6)	76 (74.5)
We monitor staff actions and the way we do things to evaluate the root cause of mistakes that happen in this pharmacy	6 (5.9)	22 (21.6)	74 (72.5)
Staff feel like their mistakes are held against them	20 (19.6)	42 (41.2)	40 (39.2)
Organizational learning and continuous improvement (PRR = 66.6)			
When a mistake occurs, we rule to sort out the problems in the work process that led to the mistake	4 (3.9)	30 (29.4)	68 (66.7)
When similar mistake keeps occurring, we make changes in the way we do things	4 (3.9)	26 (25.5)	72 (70.6)
Mistakes have led to positive changes in this pharmacy	14 (14.7)	24 (23.5)	64 (62.7)
The overall perception of patient safety (PRR = 54.9)			
This pharmacy emphasizes more on sales over patient safety	28 (27.5)	44 (43.1)	30 (29.4)
This pharmacy setup is good enough to prevent mistakes	6 (5.9)	30 (29.4)	66 (64.7)
The way we do things reflects a strong focus on patient safety in this pharmacy	2 (2.0)	28 (27.5)	72 (70.6)

Positive response rate of the participants

**Figure 1:**
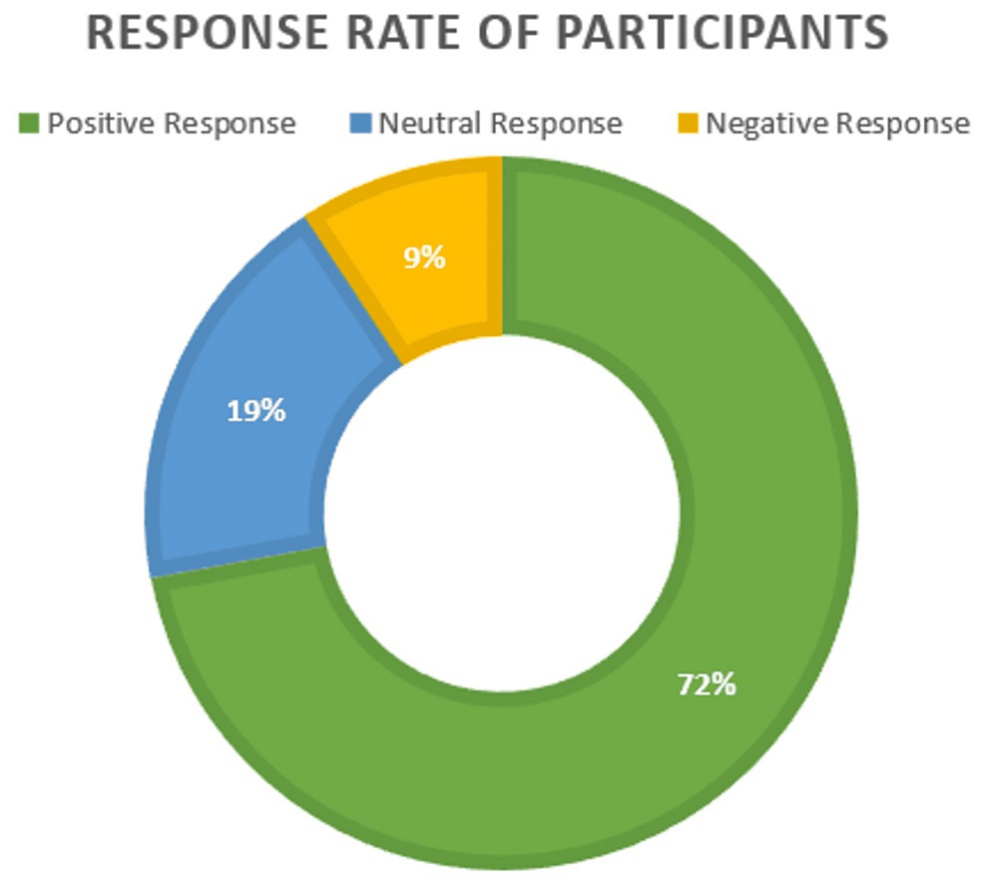
Response rate of the participants for patient safety culture (N = 102)

Figure 1 shows the overall response rate of the participants for patient safety culture. Most of the participants showed a positive response (72.1%), followed by neutral (18.6%) and negative (9.3%).

Association of demographic characteristics with patient safety culture among the participants

Table 3 presents the association of demographic characteristics with patient safety culture, specifically regarding positive, neutral, and negative responses among pharmacy staff working in different community pharmacies. While comparing different variables with the working environment in their pharmacy, a significant association was observed between age groups, professional experience, working hours per week, and position while working in the pharmacy (all p-values < 0.05). Regarding communication and work pace, a significant correlation between professional experience and working hours with communication and work pace (p-values < 0.05). Regarding patient safety and response to errors, there was a significant difference among the participants with different professional experiences (p-value < 0.001).

**Table 3:** Association of demographic characteristics with patient safety culture (N = 102) *Pharmacy technician and managerial staff

Variables	Working in this pharmacy			P-value	Communication and work pace			P-value	Patient safety and response to mistakes			P-value
	Negative	Neutral	Positive		Negative	Neutral	Positive		Negative	Neutral	Positive	
Gender												
Female	6	14	49	0.851	4	10	55	0.529	2	18	49	0.575
Male	4	6	23		4	4	25		0	10	23	
Age groups												
19-25	0	10	40	0.002	2	8	40	0.345	0	16	34	0.352
26-30	6	10	22		4	6	28		2	8	28	
>30	4	0	10		2	0	12		0	4	10	
Professional experience in the facility (years)												
<1	0	12	32	0.017	0	4	40	0.002	0	4	40	<0.001
1-3	8	4	30		6	4	32		0	14	28	
>3	2	4	10		2	6	8		2	10	4	
Working hours per week												
<30	6	6	10	0.008	0	0	22	0.031	0	6	16	0.929
31-40	0	0	6		0	0	6		0	2	4	
>40	4	14	56		8	14	52		2	20	52	
Pharmacy position												
Pharmacy intern	6	0	10	<0.001	0	0	16	0.145	0	6	10	0.588
Pharmacist	4	20	58		8	14	60		2	22	58	
Others*	0	0	4		0	0	4		0	0	4	

Information about the documentation of mistakes

The details regarding the documentation of mistakes are presented in Table 4. The participants were questioned about the frequency of mistakes and their documentation. Moreover, it was revealed that the mistakes were not regularly documented, regardless of their impact.

**Table 4:** Information about the documentation of mistakes

Mistake documentation	Always, N (%)	Never, N (%)	Sometimes, N (%)
How often is it documented when a mistake reaches the patient and could cause harm but does not?	8 (7.8)	24 (23.5)	70 (68.6)
When a mistake reaches the patient but it has no harmful potential, how often is it documented?	12 (11.7)	14 (13.7)	76 (74.6)
When a mistake that could have harmed the patient is corrected before the medication leaves the pharmacy, how often is it documented?	20 (19.6)	22 (21.5)	60 (58.9)

Overall rating on patient safety

**Figure 2:**
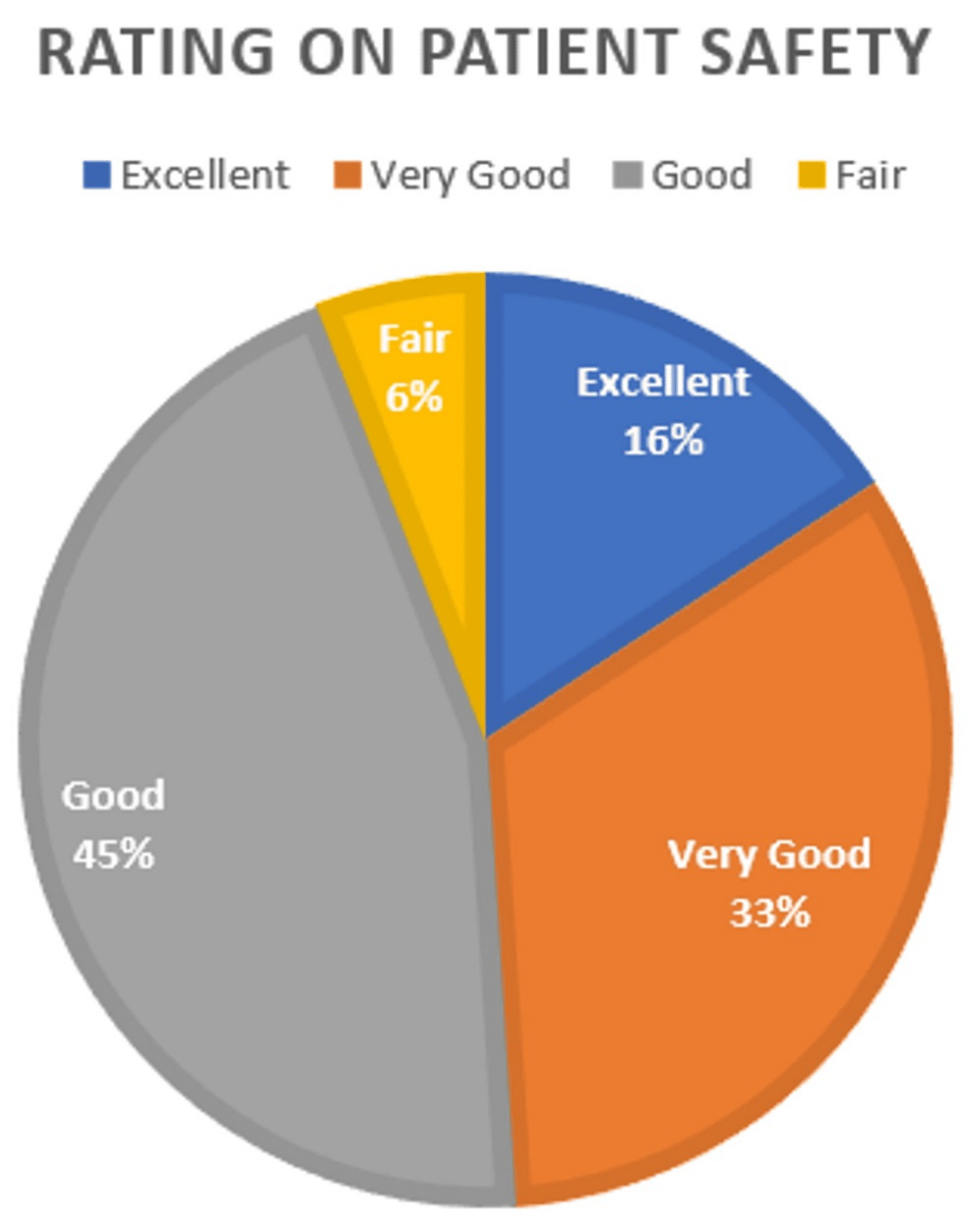
Participants' patient safety rating

The participants were asked to give ratings on patient safety at their pharmacy premises. Figure 2 represents the overall rating of patient safety. The majority of study participants responded that the level of patient safety is good (n = 46, 45.1%), followed by very good (n = 34, 33.3%), excellent (n = 16, 15.7%), and fair (n = 6, 5.9%).

## Discussion

Based on our knowledge, this study represents the first of its kind conducted in Pakistan to investigate the perceptions of pharmacy staff on PSC in community pharmacy setups. The results of our study indicated that the majority of the study participants considered the level of patient safety to be "good" (45.1%), followed by "very good" (33.3%) and "excellent" (15.7%). However, a small proportion of participants (5.9%) rated patient safety as "fair."

In contrast, a nationwide survey from Saudi Arabia reported a higher percentage of study participants (62.6%) who evaluated their pharmacy as having an "excellent" or "very good" level of patient safety [12]. Additionally, the average PRR for the 11 dimensions of the PSOPSC survey was calculated to be 60.2% [12], which is relatively lower than revealed in our study, i.e., 72.11%. This suggests that our study participants generally rated patient safety higher than those in the nationwide survey from Saudi Arabia. These differences may be attributed to variations in the study populations, healthcare settings, or other factors that could influence perceptions of patient safety. However, it is evident that our findings indicate a significant deviation, with a notably lower PPR of 72.11% when compared to the benchmark score of 82.5% derived from data provided by the AHRQ based on 479 community pharmacy staff members in the United States [13]. Therefore, the results highlight the need to enhance PSC in community pharmacies to improve patient safety.

Additionally, the findings revealed substantial inconsistency in the PPR response values across the 11 dimensions of patient safety, with "environment and physical spacing," "staffing and work pressure," "response to mistakes," "organizational learning and continuous improvement," and "overall perception of patient safety" being the dimensions with PPR below 70%. On the other hand, the highest PPR was observed with the "communication of mistakes," "communication across shifts," and "communication openness" dimensions. This implies that community pharmacies have robust communication mechanisms even across the shifts, and also, there is a culture of discussing mistakes, and staff feel comfortable suggesting and presenting their ideas. It is worth noting that the dimensions with lower PPR values indicate areas where improvements are needed to enhance patient safety. By focusing on addressing issues related to the environment, staffing, response to mistakes, organizational learning, and overall perception of patient safety, community pharmacies can work toward achieving higher levels of patient safety. Additionally, the strengths observed in communication-related dimensions can serve as examples to promote effective communication practices throughout all aspects of patient safety. Organizations that cultivate a positive safety culture prioritize communication based on mutual trust and a collective understanding of the value of patient safety [11]. In the dispensation of drugs, effective communication is crucial to minimize drug-related errors. It is imperative for pharmacy workers to actively learn from each other by gaining a comprehensive understanding of their individual roles, participating in discussions about mistakes, and providing feedback. Enhancing patient safety is greatly facilitated when pharmacy staff share a common understanding of patient safety issues, possess extensive knowledge about error sources, and utilize effective strategies for identification and prevention [14]. This can significantly enhance patient safety.

In addition, our study revealed a consistent lack of documentation regarding mistakes in community pharmacy setups, irrespective of the communication practices employed. Documentation serves as a critical tool in the prevention of medical errors and preventably enhances patient safety by preventing avoidable drug-associated harms [15]. However, the participants of our research presented an unsatisfactory attitude toward documenting mistakes. The lack of standardized systems inside pharmacies could be an obstacle to effective documentation. It is essential to make significant efforts to promote a culture of reporting and documentation to enhance patient safety in community pharmacies.

The composites "overall perceptions of patient safety" and "response to mistakes" exhibited the lowest PPR, with values of 54.9% and 58.1%, respectively. Consistent with the findings of Westat [16], the current study reported that around 39.2% of the participants felt like their mistakes were held against them, and 60% responded that they were treated fairly for committing the mistakes. Furthermore, around 75% of the participants were inclined to monitor staff actions and understand why mistakes occur. It is crucial to conduct a root cause analysis in the event of a patient safety incident. When conducting root cause analysis, a systematic exploration is employed to uncover the underlying causes and environmental context in which the incident occurred, extending beyond the individuals implicated [17]. The application of this approach is commonly employed in healthcare institutions to learn from system failures resulting from clinical incidents and identify patient safety improvement initiatives that can successfully eliminate or control the risks associated with these events [18-20].

The pharmacy environment notably influences factors associated with medication errors [21-23]. Studies indicate that an unfavorable working environment is often linked to high rates of dispensing errors [24,25]. In accordance with the findings of our study, the composite "environment and physical spacing" had a low score in the studies conducted in China, Kuwait, Malaysia, Qatar, and the United States [13,16,26-28]. A significant number of our participants highlighted that distractions and interruptions in the pharmacy setting pose challenges for staff to work with precision. Based on these findings, providing community pharmacists with a supportive and conducive workplace environment is crucial to enhance their efficiency.

A potential drawback of using questionnaires as a data collection method is the possibility of response bias, as the participants may respond in a way that is influenced by their biases or perceptions [29].

Firstly, our study employs a convenience sampling method, which could introduce selection bias. This means that the participants may not be fully representative of all community pharmacies in Karachi, potentially limiting the external validity of our results. The sample may not capture the diversity that exists within the community pharmacy landscape, and caution should be exercised when extending our findings to the broader population.

Secondly, the relatively small sample size may impact the statistical power of our analysis. A smaller sample size might limit our ability to detect subtle but potentially meaningful associations between patient safety culture and the demographic characteristics of pharmacy staff. The findings should be interpreted with this limitation in mind, recognizing that larger-scale studies may be needed to corroborate and strengthen the evidence presented here.

Despite these limitations, our study provides valuable insights into the patient safety culture of community pharmacies in Karachi. Recognizing these constraints, future research endeavors should aim for more extensive and diverse sampling methods to enhance the generalizability of findings and contribute to a more comprehensive understanding of patient safety in community pharmacy settings.

Future research could benefit from including a question to distinguish the participants' professional backgrounds for a more nuanced analysis and accurate interpretation of survey results in this context. Moreover, research may employ a triangulation (observation) approach in parallel to a survey questionnaire to validate the findings from the survey. Further, allowing the comparisons of study findings among individuals or chains of pharmacies; pharmacy working hours, i.e., >40 hours/week or <40 hours/week; and work experience may expand the insights.

## Conclusions

The research findings illustrated that the pharmacies under study in Karachi, Pakistan, have developed strategies to address communication barriers that hinder effective communication between pharmacy staff and patients. However, weaknesses were identified in domains such as staffing, work pressure, teamwork, and response to mistakes. Enhancing these areas would contribute to an improved perception of patient safety. Additionally, there is a pressing need to raise awareness about the importance of documentation and reporting and allocate appropriate staff resources to alleviate pressure.

## References

[1] AlThubaity DD, Mahdy Shalby AY (2023). Perception of health teams on the implementation of strategies to decrease nursing errors and enhance patient safety. J Multidiscip Healthc.

[2] (2017). Patient safety: making health care safer. https://www.who.int/publications/i/item/WHO-HIS-SDS-2017.11.

[3] Nieva VF, Sorra J (2003). Safety culture assessment: a tool for improving patient safety in healthcare organizations. Qual Saf Health Care.

[4] Hong K, Hong YD, Cooke CE (2019). Medication errors in community pharmacies: the need for commitment, transparency, and research. Res Social Adm Pharm.

[5] Phipps DL, Jones CE, Parker D, Ashcroft DM (2018). Organizational conditions for engagement in quality and safety improvement: a longitudinal qualitative study of community pharmacies. BMC Health Serv Res.

[6] Phipps DL, Noyce PR, Parker D, Ashcroft DM (2009). Medication safety in community pharmacy: a qualitative study of the sociotechnical context. BMC Health Serv Res.

[7] Atif M, Razzaq W, Mushtaq I, Malik I, Razzaq M, Scahill S, Babar ZU (2020). Pharmacy services beyond the basics: a qualitative study to explore perspectives of pharmacists towards basic and enhanced pharmacy services in Pakistan. Int J Environ Res Public Health.

[8] Atif M, Malik I (2020). COVID-19 and community pharmacy services in Pakistan: challenges, barriers and solution for progress. J Pharm Policy Pract.

[9] Wazir MA, Goujon A (2019). Assessing the 2017 census of Pakistan using demographic analysis: a sub-national perspective. Papers.

[10] Pervez S, Jabbar AA, Haider G (2020). Karachi Cancer Registry (KCR): age-standardized incidence rate by age-group and gender in a mega city of Pakistan. Asian Pac J Cancer Prev.

[11] Franklin M, Sorra J (2013). Community pharmacy survey on patient safety culture. https://www.ahrq.gov/sops/surveys/pharmacy/index.html.

[12] Almalki ZS, Alshehri AM, Alturki LA (2021). Exploring patient-safety culture in the community pharmacy setting: a national cross-sectional study. Postgrad Med.

[13] Jia P, Zhang L, Zhang M (2014). Safety culture in a pharmacy setting using a pharmacy survey on patient safety culture: a cross-sectional study in China. BMJ Open.

[14] Alsaleh FM, Abahussain EA, Altabaa HH, Al-Bazzaz MF, Almandil NB (2018). Assessment of patient safety culture: a nationwide survey of community pharmacists in Kuwait. BMC Health Serv Res.

[15] Sivanandy P, Maharajan MK, Rajiah K, Wei TT, Loon TW, Yee LC (2016). Evaluation of patient safety culture among Malaysian retail pharmacists: results of a self-reported survey. Patient Prefer Adherence.

[16] Westat R (2012). 2012 preliminary comparative results: pharmacy survey on patient safety culture. Services.

[17] Hu M, Yee G, Zhou N, Yang N, Jiang X, Klepser D (2014). Development and current status of clinical pharmacy education in China. Am J Pharm Educ.

[18] Mackinnon GE III, Mackinnon NJ (2008). Documentation of pharmacy services. Pharmacotherapy: a pathophysiologic approach.

[19] Owusu YB, Abouelhassan R, Awaisu A (2021). Evaluation of patient safety culture in community pharmacies in Qatar. Int J Clin Pract.

[20] Knudsen P, Herborg H, Mortensen AR, Knudsen M, Hellebek A (2007). Preventing medication errors in community pharmacy: root-cause analysis of transcription errors. BMJ Qual Saf.

[21] Hooker AB, Etman A, Westra M, Van der Kam WJ (2019). Aggregate analysis of sentinel events as a strategic tool in safety management can contribute to the improvement of healthcare safety. Int J Qual Health Care.

[22] Nicolini D, Waring J, Mengis J (2011). Policy and practice in the use of root cause analysis to investigate clinical adverse events: mind the gap. Soc Sci Med.

[23] Charles R, Hood B, Derosier JM (2016). How to perform a root cause analysis for workup and future prevention of medical errors: a review. Patient Saf Surg.

[24] Pervanas HC, Revell N, Alotaibi AF (2016). Evaluation of medication errors in community pharmacy settings: a retrospective report. J Pharm Technol.

[25] Shahrokhi A, Ebrahimpour F, Ghodousi A (2013). Factors effective on medication errors: a nursing view. J Res Pharm Pract.

[26] Nguyen EE, Connolly PM, Wong V (2010). Medication safety initiative in reducing medication errors. J Nurs Care Qual.

[27] Aldhwaihi K, Schifano F, Pezzolesi C, Umaru N (2016). A systematic review of the nature of dispensing errors in hospital pharmacies. Integr Pharm Res Pract.

[28] Alam S, Osama M, Iqbal F, Sawar I (2018). Reducing pharmacy patient waiting time. Int J Health Care Qual Assur.

[29] Smith F (2010). Conducting your pharmacy practice research project: a step-by-step approach. Pharmaceutical press.

